# Supplementary material for: A Critical View of the Application of the APEX Software (Aqueous Photochemistry of Environmentally-Occurring Xenobiotics) to Predict Photoreaction Kinetics in Surface Freshwaters
Source: Molecules. 2019 Dec 18;25(1):9. doi: 10.3390/molecules25010009 (PMC7017383; doi:10.3390/molecules25010009)
Supplement: Supplementary file 1 [file molecules-25-00009-s001.zip › 3.2.4_gcc-4.4.0.zip!/3.2.4_gcc-4.4.0/3.2.4_gcc-4.4.0/doc/octave/html/interpreter/Comment-Tips.html]

Comment Tips - Untitled


Next: Function Headers,
Previous: Coding Tips,
Up: Tips and Standards


---

### C.3 Tips on Writing Comments

Here are the conventions to follow when writing comments.

#: Comments that start with a single sharp-sign, #, should all be aligned to the same column on the right of the source code. Such comments usually explain how the code on the same line does its job. In the Emacs mode for Octave, the `M-;` (`indent-for-comment`) command automatically inserts such a # in the right place, or aligns such a comment if it is already present. ##: Comments that start with a double sharp-sign, ##, should be aligned to the same level of indentation as the code. Such comments usually describe the purpose of the following lines or the state of the program at that point.

The indentation commands of the Octave mode in Emacs, such as `M-;`
(`indent-for-comment`) and `TAB` (`octave-indent-line`)
automatically indent comments according to these conventions,
depending on the number of semicolons. See Manipulating Comments (The GNU Emacs Manual).
